# From appressorium to transpressorium—Defining the morphogenetic basis of host cell invasion by the rice blast fungus

**DOI:** 10.1371/journal.ppat.1009779

**Published:** 2021-07-30

**Authors:** Neftaly Cruz-Mireles, Alice Bisola Eseola, Míriam Osés-Ruiz, Lauren S. Ryder, Nicholas J. Talbot

**Affiliations:** The Sainsbury Laboratory, University of East Anglia, Norwich Research Park, Norwich, United Kingdom; Geisel School of Medicine at Dartmouth, UNITED STATES

## Introduction

To cause disease, many fungal pathogens develop specialised structures to rupture the tough outer layers of their plant or animal hosts. These infection cells, called appressoria, have been extensively studied in many fungal species [1]. However, once inside host tissue, pathogens must also invade new cells and traverse host cell junctions. How they do this has received much less attention, but recent evidence from the rice blast fungus suggests that cell invasion within a host plant may also require the development of a specialised infection structure. Here, we compare the developmental biology of invasive growth during different stages of plant infection by the rice blast fungus. We identify the remarkable parallels between the biology of appressorium development and cell-to-cell movement. Finally, we evaluate evidence suggesting that a specialised infection cell—the transpressorium—is necessary for invasive growth.

### How does the rice blast fungus puncture an intact leaf?

Rice blast disease is one of the world’s most important crop diseases, each year destroying enough rice to feed 60 million people [2]. Given that rice is the staple food for almost 3.7 billion of the world’s population—many of them in low-income countries—blast disease represents a clear and present danger to global food security. The blast fungus *Magnaporthe oryzae* can, however, infect more than 50 different grass species, including other major cereals such as barley, oats, finger millet, and wheat. Significant outbreaks of wheat blast have occurred in Brazil, Bangladesh [3,4], and, most recently, in Zambia [5]—now threatening wheat production on 3 continents. Understanding the biology of blast diseases is therefore important if new disease control strategies are to be developed.

To gain entry to a plant, *M*. *oryzae* uses a dome-shaped, melanin-pigmented appressorium [1]. A conidium germinates on the leaf surface to form a polarised germ tube, which differentiates into an appressorium within 4 to 6 hours. There are 3 important prerequisites for appressorium morphogenesis. First of all, a hard hydrophobic surface [6] must be recognised by *M*. *oryzae*, which requires the Pmk1 mitogen-activated protein kinase (MAPK) signalling pathway. Upstream sensory proteins trigger a phosphorylation cascade that involves Mst11 (mitogen-activated protein kinase kinase kinase, MAPKKK), Mst7 (mitogen-activated protein kinase kinase, MAPKK), and Pmk1 (MAPK). In the absence of Pmk1, the fungus is unable to form an appressorium, and, therefore, incapable of causing disease [7]. The second prerequisite is that the germinating cell of the 3-celled conidium must undergo mitosis. An S phase checkpoint is necessary for the initiation of appressorium development, and the nucleus must then pass through G2-M to enable appressorium maturation to progress [8]. Finally, the 3-celled conidium undergoes autophagy and an iron-dependent programmed cell death process, called ferroptosis, before its contents are trafficked to the appressorium [9,10]. If this process is impaired by mutation of genes required for autophagy, then the fungus is unable to cause disease because appressoria cannot repolarise. The initiation of autophagy requires both Pmk1 and cell cycle progression, but is also linked to starvation stress and a target of rapamycin (TOR) kinase–dependent metabolic checkpoint [11], because appressoria only develop in the absence of exogenous nutrients.

### How does the appressorium function?

Once formed, the appressorium adheres tightly to the leaf cuticle and develops enormous turgor of up to 8.0 MPa (approximately 80 atmospheres of pressure). This huge pressure is generated by accumulating high concentrations of glycerol and other polyols [12,13], which draw water into the cell by osmosis. The appressorium has a differentiated cell wall rich in melanin, which reduces cell wall porosity, thereby preventing exodus of polyols but allowing water entry to continue. Melanisation of the appressorium is essential for turgor generation, and mutants that cannot synthesise dihydroxynaphthalene melanin are unable to cause blast disease [14]. Turgor is applied at the base of the appressorium as mechanical force, enabling a narrow, rigid penetration hypha to rupture the rice leaf cuticle [13]. This requires cytoskeletal reorientation, followed by rapid actin polymerisation [15–17]. Filamentous actin forms a toroidal network around the appressorium pore, a region at the base of the appressorium lacking melanin, which marks the points from which the penetration peg emerges [15,18]. Septin guanosine triphosphatases (GTPases) are necessary for actin remodelling, forming a ring structure around the appressorium pore, which provides cortical rigidity and acts as a lateral diffusion barrier. This facilitates the organisation of polarity determinants and proteins involved in membrane deformation and exocytosis [15,17]. In the absence of any of the 4 core septins that form the hetero-oligomeric septin ring at the appressorium pore, the cell is unable to repolarise and puncture the leaf cuticle. Penetration peg emergence therefore involves a switch from isotropic to polarised, anisotropic growth at the appressorium pore [19]. It is also clear that these changes in cytoskeletal conformation only occur once a critical threshold of appressorium turgor has been achieved [20]. A turgor-sensing histidine-aspartate kinase, Sln1, is necessary for sensing when maximal turgor has been reached, modulating further pressure generation. Mutants lacking the Sln1 kinase generate excess appressorial turgor, but cannot repolarise and are thus unable to apply the pressure generated as protrusive force [20]. Sln1 is necessary to down-regulate both glycerol synthesis, likely regulated by the cAMP-dependent protein kinase A pathway, and melanisation. As a consequence, *sln1* mutants form hypermelanised nonfunctional appressoria [20]. In addition, a pressure-dependent cell cycle S phase checkpoint in the appressorium is essential for septin-dependent repolarisation [21].

### How is rice tissue colonised by the blast fungus?

**Fig 1:**
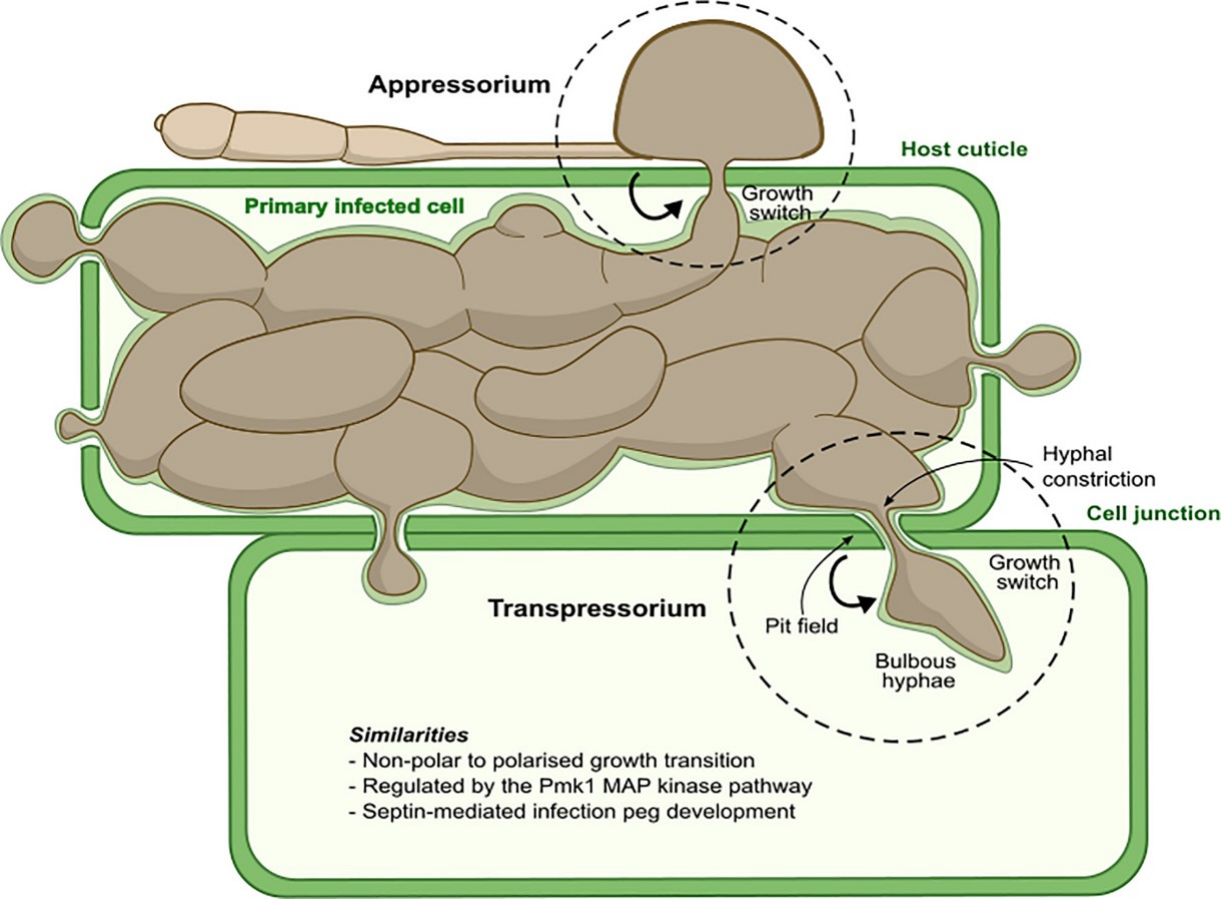
Infection structures of the rice blast fungus: The appressorium and transpressorium. A schematic figure describing the characteristics and developmental biology of appressoria and transpressoria—specialised infection structures formed by *Magnaporthe oryzae* to penetrate the host cuticle and traverse cell junctions, respectively. MAP, mitogen-activated protein.

Once inside a plant cell, the penetration hypha differentiates into bulbous, branched hyphae that rapidly fill the interior of the cell. These invasive hyphae grow by budding, and the fungus undergoes significant changes in primary metabolism [22] during initial cell colonisation. Soon after its entry into a plant cell, a plant membrane–rich cap is also observed at the tip of the penetration peg. The fungus buds at this point and differentiates into an invasive hypha, but the membrane-rich structure remains and is known as the biotrophic interfacial complex (BIC) [23,24]. The BIC might originate as a focal plant defence reaction, but an increasing body of evidence suggests that *M*. *oryzae* utilises this structure to deliver effector proteins into plant cells. Effectors are secreted pathogen proteins necessary for suppression of plant immune responses. Secretion of effectors into the cytoplasm involves a distinct secretory pathway to conventional hyphal tip–mediated secretion of extracellular effectors [24]. Only once the fungus has fully occupied the initial epidermal cell does it invade adjacent cells, normally in a highly synchronous manner, spreading from cell to cell and rapidly occupying host tissue (Fig 1).

### What is a transpressorium?

**Fig 2:**
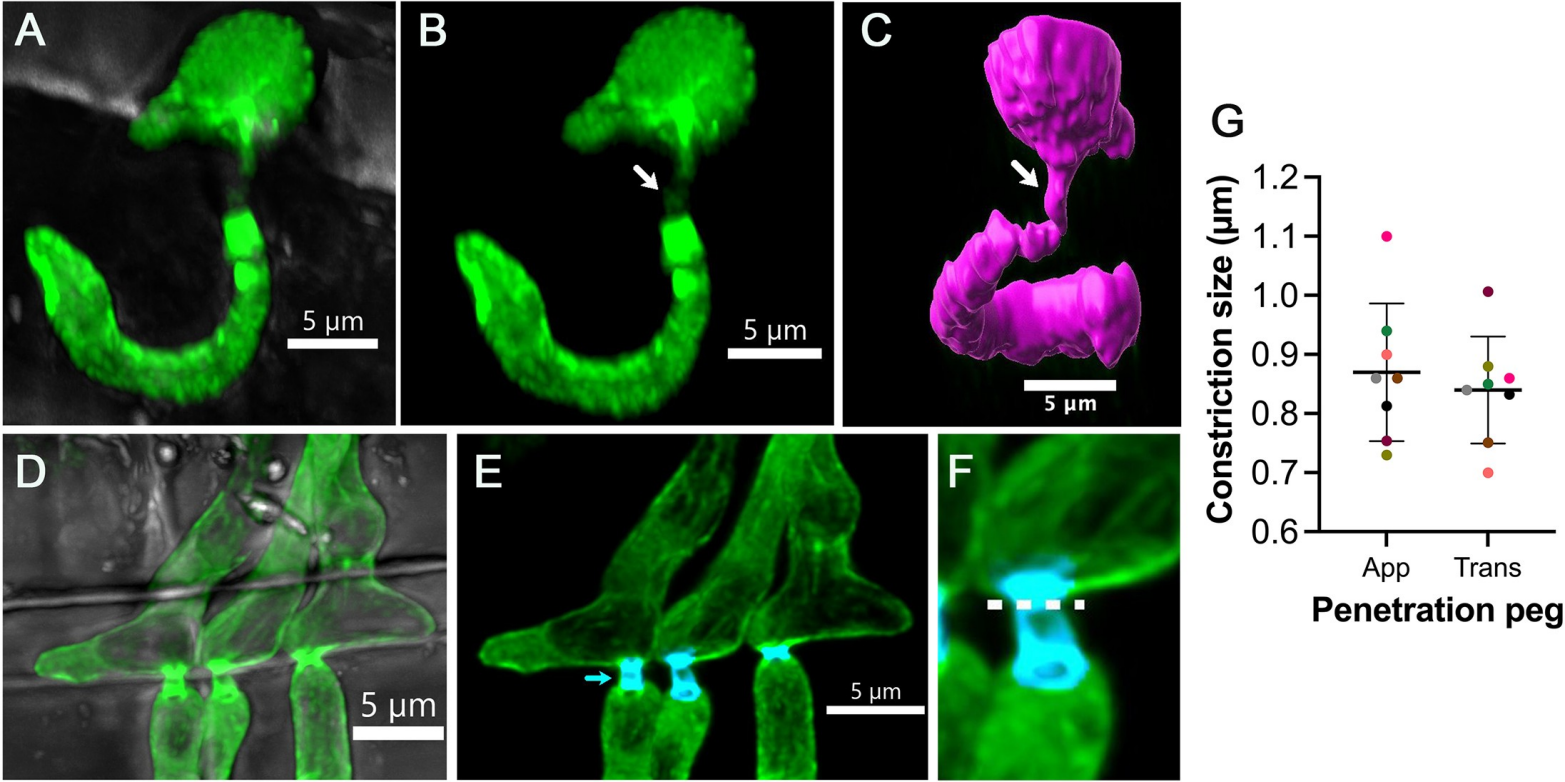
Cell-to-cell movement during the rice blast infection. Live-cell imaging of *Magnaporthe oryzae* strain expressing gelsolin–GFP during rice infection. **(A, B)** Three-dimensional projection micrographs showing the appressorium entry site into rice leaf sheath. The arrow indicates the penetration peg, which subsequently differentiates into a primary invasive hypha at 24 hpi. **(C)** Three-dimensional rendering of the base of the *M*. *oryzae* appressorium with a penetration peg (arrowed) and primary invasive hypha within an epidermal rice cell. **(D, E)** Three-dimensional projection micrographs to show the specialised transpressorium required for cell-to-cell invasion by *M*. *oryzae*. The cyan colour indicates the constriction site when the fungus passes through a plasmodesmata-rich pit field. **(F)** Enlarged image of the transpressorium crossing point. The dotted line indicates the region selected for measurement of the diameter of the hyphal constriction point. **(G)** Plot showing the mean diameter of the App and Trans. Data were collected from 3 different rice seedling infections (*n* = 8 pegs measured in each experiment), with data points colour coded for each biological replication of the experiment. App, appressorium penetration peg; Trans, transpressorium penetration peg.

How fungal pathogens spread from cell to cell in host tissue is largely unstudied in either plant or animal pathogenic fungi. In *M*. *oryzae*, severe hyphal constrictions were observed during invasive growth and appeared to correlate with pit fields where plasmodesmata accumulate [23]. Plasmodesmata are cytoplasmic conduits that link together plant cells [25]. Live-cell imaging of cell-to-cell movement by *M*. *oryzae* has shown that invasive hyphae become swollen (approximately 5.0 μm in diameter) at rice cell junctions and then undergo severe hyphal constriction to a diameter of 0.6 to 0.8 μm (measured by electron microscopy) [26]. This is very similar to the process that occurs when an appressorium forms a penetration peg, with both structures having a similar diameter when visualised by light microscopy (0.8 to 0.9 μm), as shown in Fig 2. Hyphal constriction is accompanied by actomyosin ring formation at the cell junctions. Interestingly, it has been reported that the Pmk1 MAPK cascade, which regulates appressorium morphogenesis, is also necessary for hyphal constriction and cell-to-cell invasion in a septin-dependent mechanism [26]. Using a conditional analogue–sensitive mutant of Pmk1, it was shown that the inhibition of the Pmk1 MAPK with the ATP analogue Napthyl-PP1 prevents *M*. *oryzae* from moving between rice cells [26]. This suggests that the Pmk1 pathway is involved in the morphogenetic switch of bulbous hyphae into narrow infection pegs that traverse rice cells. Interestingly, Pmk1 also regulates the expression of a subset of fungal effector genes that may be required for suppression of plasmodesmatal immunity. During initial infection, plasmodesmatal conductance is maintained to enable effectors to move into adjacent unoccupied plant cells, which may involve manipulation of plasmodesmata by fungal effectors to prevent their closure. However, it is also clear that even when plant immune responses are suppressed or host tissue is killed, the fungus still needs to undergo pit field location and hyphal constriction in order to traverse cell junctions [26].

Appressorium development and cell-to-cell movement therefore appear to be morphogenetically related processes. Both involve isotropic expansion of a swollen germ tube or a hyphal tip, followed by the generation of a much narrower infection peg, to rupture either the cuticle or plant cell wall at pit fields. This similarity has been noted previously by careful observers of plant–fungal interactions, who coined the term “transpressorium” to describe *in planta* infection structures formed by fungi to move between host cells. Liese and Schmid were the first to describe transpressoria when they studied *Ceratocystis* sp. infection of *Pinus strobus* [27,28]. They reported how swollen structures underwent severe constriction to form hyphae of much smaller diameter involved in the penetration of the cell wall of neighbouring cells [27]. Once the transpressorium peg reached the lumen of the adjacent cell, it then expanded to the normal diameter of an invasive hypha [27]. Liese suggested that the transpressorium penetrates the cell wall using a combination of localised enzymatic activity and mechanical pressure [29]. Appressoria and transpressoria therefore fulfil a very similar function, enabling traversal of a physical obstacle [27,30]. Although these findings were reported more than 55 years ago, there have not been further reports of transpressorium morphogenesis. Recent observations in *M*. *oryzae* of its Pmk1-dependent, septin-mediated cell-to-cell movement mechanisms, however, are completely consistent with the experiments of Liese and Schmid [27] and thoughtful reviewers of appressorium biology [28].

Transpressorium-like structures have also been reported in other filamentous fungi. Hyphal morphogenetic reprogramming into specialised structures has been largely studied in model fungal species, such as *Neurospora crassa* and *Aspergillus nidulans* [31]. Recent studies in *Podospora anserina* have, for example, shown that narrow hyphae are developed during fungal growth in order to breach cellulosic substrates such as cellophane [32]. Additionally, recent elegant studies of hyphal morphological adaptation to occupy extremely narrow channels suggest that a trade-off may exist between plasticity and velocity in hyphal growth [33]. These observations provide evidence that generation of specialised hyphae-derived structure for invasive growth may be a conserved mechanism in filamentous fungi. Elucidating the common morphological components of transpressorium and transpressorium-like invasive hyphae will be an exciting future challenge.

### What are the parallels between appressoria and transpressoria?

Many common features are shared between appressoria and transpressoria. First, their development involves departure from polarised growth and formation of an isotropically expanded hyphal/germ tube tip. Both types of infection cell also form following recognition of physical cues of the surfaces they encounter [34]. A symmetry breaking process then occurs, whereby a polarised infection peg is formed to rupture the host cell wall, either at the leaf surface or at pit fields between host cells. Finally, after passing through the structural barrier, the emerging infection hypha is surrounded by the invaginated plant plasma membrane. This occurs not only upon initial infection, but also, remarkably, every time the fungus enters a new host cell [26]. A separate extra-invasive hyphal membrane compartment is always formed as well as a BIC [23,26]. These morphogenetic processes during both appressorium and transpressorium development require the Pmk1 MAPK—acting downstream of the thigmotropic perception of the cell/cuticle surface—which regulates septin-dependent cytoskeletal remodelling.

### What do we not understand about invasive growth by the blast fungus?

The obvious parallels between appressorium and transpressorium development raise many questions. What are the thigmotropic signals, for example, perceived by hyphal tips, which lead to appressorium and transpressorium morphogenesis, and which sensory proteins are necessary for their perception? While some putative sensors have been identified for appressorium development [35,36], this process is far from well understood. Does perception of these surface cues lead to membrane curvature generation in the fungus, acting as a signal for septin aggregation during development of infection cells [37,38]? This might, for example, explain how pit fields are recognised as indentations in the cell wall surface. Is there a cell cycle dependency for transpressorium development, as there is for appressorium formation? Mitosis occurs at cell junctions [39], but is this a prerequisite for transpressorium formation, and, if so, does a similar S phase checkpoint mechanism act at this time [21]? Is there a quorum sensing or nutritional dependency for transpressorium development? Invasive hyphae appear to fill epidermal cells completely before invasion of neighbouring cells, suggesting that such a signal might exist, while it is also clear that transpressorium function and biotrophic growth may be linked to metabolic control and TOR kinase regulation [40]. Finally, and perhaps most intriguing of all, does transpressorium function require pressure generation and application of mechanical force in the same way as an appressorium, and, if so, are transpressoria ever melanised? Or, alternatively, does cell wall crossing occur exclusively via enzymatic activity?

We have much to learn about the mechanisms of invasive growth by pathogenic fungi, but the role of the transpressorium—which has hitherto been largely unrecognised—may prove to be as significant to fungal pathogenesis as that of the appressorium.
